# Arginine-Functionalized Thin Film Composite Forward Osmosis Membrane Integrating Antifouling and Antibacterial Effects

**DOI:** 10.3390/membranes13090760

**Published:** 2023-08-28

**Authors:** Yichen Chen, Wenmeng Yu, Hu Cao

**Affiliations:** 1 School of Environment, Renmin University of China, Beijing 100872, China; cyc2020@ruc.edu.cn; 2 Rural Energy & Environment Agency, Ministry of Agriculture and Rural Affairs of the People’s Republic of China, Beijing 100125, China

**Keywords:** forward osmosis, chemical modification, wastewater treatment, antifouling, antibacterial

## Abstract

Membrane fouling is an inevitable obstacle of polyamide composite forward osmosis (FO) membranes in oily wastewater treatment. In this study, zwitterionic arginine (Arg) is grafted onto nascent self-made FO polyamide poly(ether sulfone) (PA-PES) membrane, imparting superior hydrophilic, antifouling, and antibacterial properties to the membrane. Detailed characterizations revealed that the Arg-modified (Arg-PES) membrane presented obviously surface positively charged and unique morphology. Results showed that our strategy endowed the optimized membrane, the water flux increased by 113.2% compared to the pristine membrane, respectively, meanwhile keeping high NaCl rejection > 93.9% (with DI water as feed solution and 0.5 M NaCl as draw solution, FO mode). The dynamic fouling tests indicated that the Arg-PES membranes exhibited much improved antifouling performance towards oily wastewater treatment. The flux recovery ratios of the membrane were as high as 92.0% for cationic emulsified oil (cetyl pyridinium chloride, CPC), 87.0% for neutral emulsified oil (Tween-80), and 86.0% for anionic emulsified oil (sodium dodecyl sulfate, SDS) after washing, respectively. Meanwhile, the Arg-PES membranes assembled with guanidine cationic groups exhibited an enhanced antibacterial property against *E. coli*, which exhibited a high antibacterial efficiency of approximately 96%. Consequently, the newly arginine functionalized FO membrane possesses impressive antifouling performance, while simultaneously resisting bacterial invasion, thus rendering it an ideal alternative for oily wastewater treatment in the FO process.

## 1. Introduction

The pervasive issue of oil contaminants, especially emulsified oil in wastewater, stands as a prominent environmental concern across diverse spheres encompassing our daily life and many industries, such as food, textiles, steel, mining, and petrochemical [1,2]. Treating oily wastewater to comply with regulatory discharge requirements remains a formidable challenge [3]. Conventional modalities for treating oily wastewater typically include physical adsorption, gravity separation, and centrifugation techniques [4,5]. While these methods are effective in separating suspended oil in water, they have limited effectiveness in segregating emulsified oil due to the diminutive scale of oil droplets (less than 1 μm) in such mixtures [6]. In this landscape, membrane technology emerges as a compelling prospect, characterized by its elevated separation efficiency, diminished energy consumption, and extensive scope of applicability. Traditional membrane technologies, such as microfiltration (MF), ultrafiltration (UF), and reverse osmosis (RO), predominantly rely on elevated pressure differentials for the processing of oily wastewater. However, this operational modality precipitates the adhesion of oil droplets onto membrane surfaces or pores, thereby resulting in rapid water flux attenuation and the onset of severe fouling [6,7,8]. The development of oil–water separation applications within membrane separation technology has encountered an intricate obstacle [9,10]. Consequently, forward osmosis (FO), an osmotic pressure-driven membrane technology with low fouling and reduced energy consumption, is being used to overcome such problems [11,12,13].

In the realm of FO-based oily wastewater treatment, membrane fouling remains a principal impediment that requires mitigation [14,15]. To address this gap, one of the most common strategies is to enhance the hydrophilicity of the membrane. This involves grafting hydrophilic materials onto membranes through mechanisms such as free radical reactions, UV irradiation, or esterification processes, thereby bolstering the membrane antifouling performance [16]. The aim is to achieve ultra-low or even negligible oil adhesion on the membrane surface, culminating in effective resistance against oil-induced fouling and the realization of heightened separation selectivity. However, these methods are not devoid of limitations: (1) the surface grafting can be incomplete; (2) the synthesis of the modifier is complicated, demanding harsh conditions; (3) the by-products generated during the surface grafting may inadvertently harm the integrity of membrane structure. In response to these challenges, we propose a simple, green, and efficient one-pot synthesis method, predicated on the covalent attachment of a hydrophilic material endowed with robust hydration capabilities. This results in the establishment of a repellent boundary layer through a mild amidation reaction [17].

Meanwhile, the domain of FO-based wastewater treatment confronts the enduring predicament of biofouling, which engenders adverse implications on membrane longevity and operational efficacy [18]. Biofouling materializes as bacteria, biological macromolecules, or microorganisms deposit on the membrane surface, resulting in the formation of a bacterial bilayer layer that ultimately impairs the membrane structure, curtails separation efficacy, and escalates operational expenses [19]. Various strategies have been proposed to address this issue, including feed solution pretreatment, surface modification, and chemical cleaning [20]. Despite displaying certain efficacy in enhancing the antibacterial attributes of FO membranes, the self-replicating nature of bacteria poses a challenge to their comprehensive eradication.

Accordingly, endeavors to bolster the antibacterial properties of membrane materials have yielded diverse approaches, ranging from surface coatings and the incorporation of antibacterial nanoparticles (NPs) to the reinforcement of zwitterionic polymers [19,21,22]. However, these methods have drawbacks. For example, the surface coating is prone to detachment during prolonged operation due to their reliance on weak molecular interactions. Nonetheless, antibacterial materials such as silver and copper nanoparticles are widely used for membrane preparation [23]. Furthermore, the uncontrolled release mechanism of nanoparticles leaching from the membrane surface may lead to a gradual loss of their antibacterial function and potential water safety risk [22].

In light of these considerations, attaching antibacterial materials covalently onto the surface is more conducive for FO membranes with stability, high antibacterial capacity, and minimizing environmental risks [24]. Zwitterionic polymers, typified by cationic quaternary ammonium (N+) groups, such as phosphobetaine, sulfobetaine, carboxybetaine, etc., have benign antibacterial properties and have been the primary choice for the construction of antibacterial membrane surfaces [18,25]. Despite their efficacy, the complex synthetic, tedious grafting process, and high cost make the preparation of antibacterial FO membranes problematic. Hence, it is necessary to develop a novel FO membrane that combines hydrophilicity, antifouling properties, and antibacterial capabilities via an economical and easy-to-handle method for specifically treating oily wastewater.

**Figure 1:**
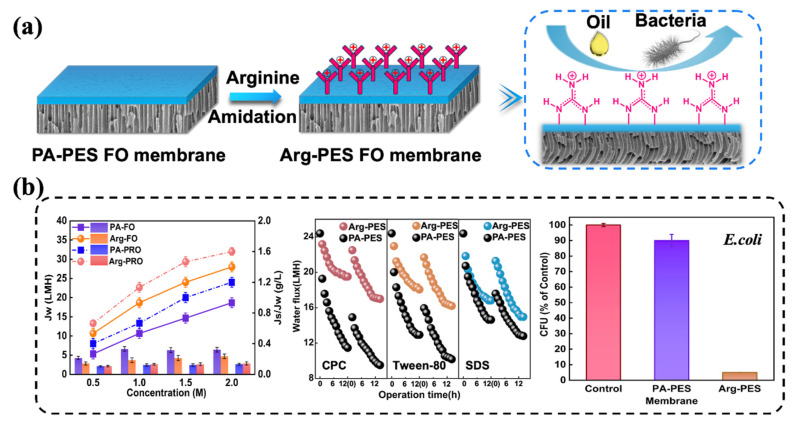
(**a**) Synthesis processes of the Arg-PES membrane. (**b**) Arginine modifier endows the membrane with superior water recovery efficiency, stronger fouling resistance, renewability, and stronger antibacterial properties.

In this study, we embark on a comprehensive exploration addressing the dual quagmires of membrane fouling and biofouling endemic to oily wastewater treatment. Taking into consideration the current unsatisfactory and costly modification methods, we developed a simple yet effective arginine (Arg)-modified polyamide (PA) polyethersulfone (PES) membrane, named the Arg-PES membrane. As shown in Figure 1, by covalently attaching hydrophilic arginine onto the membrane surface, its hydrophilicity, antifouling performance, and antibacterial ability were enhanced. Furthermore, due to the unique guanidine structure of arginine, the modified membrane exhibited cleaning and regeneration capabilities. We conducted tests and analyses on both unmodified and modified membranes regarding their surface chemical structure, hydrophilicity, morphology, roughness, zeta potential, as well as separation performance. Additionally, three emulsified oils formed by different types of surfactants (cetyl pyridinium chloride (CPC), Tween-80, and sodium dodecyl sulfate (SDS)) were used to evaluate the antifouling properties and regeneration effect of the membranes. Finally, the antibacterial performance was assessed using Escherichia coli (*E. coli*).

## 2. Materials and Methods

### 2.1. Materials and Chemicals

Arg was obtained from Adamas. All the chemicals used were of analytical grade or the highest grade available. PES (Solvay Advanced Polymer, L.L.C, Alpharetta, GA, USA), n-methyl-2-pyrrolidone (NMP, Aladdin, Shanghai, China), and polyethylene glycol with a molecular weight (MW) of 400 (PEG 400, Fuchen, Tianjin, China) were used to prepare dope solutions for membrane substrate fabrication. Trimesoyl chloride (TMC, Micxy, Chengdu, China), m-phenylenediamine (MPD, Aladdin), and n-hexane (Fisher Scientific, Waltham, MA, USA) were deployed to synthesize the PA selective layer on the top of either PES substrate. Reagents of NaOH (99%) and HCl (98%) supplied by Tianjin Fuchen Chemical Reagents Co., Ltd. (Tianjin, China) were used to adjust the pH values of solution. PEG with MW = 12,000 and 35,000 g·mol^−1^, polyethylene oxide (PEO) with MW = 100,000 and 200,000 g·mol^−1^ from Sigma-Aldrich (St. Louis, MO, USA) were deployed to characterize the molecular weight cut-off (MWCO), mean pore size, and pore size distribution of PES substrates. Ethylene glycol, diethylene glycol, triethylene glycol, and glucose purchased from Fuchen Chemical Reagent Co., Ltd. (Tianjin, China) were used to characterize the structure properties of FO membrane. NaCl (Fuchen) served as a draw solute for FO experiments. Petroleum (Aladdin), CPC, Tween-80, and SDS were ordered from Adamas to prepare the oil-in-water emulsion, respectively. Escherichia coli (*E. coli*), Luria-Bertani (LB) broth, and Bacto agar purchased from Qingdao Hope Bio-Technology Co., Ltd. (Qingdao, China) were used for antibacterial experiments. DI water produced from an ultrapure water system (Millipore, Burlington, MA, USA) with a resistivity of 18.25 MΩ·cm was used in all experiments.

### 2.2. Preparation of the PA-PES FO Membrane

A polymer solution containing 42.5 wt% PEG 400, 15.0 wt% PES, and 42.5 wt% NMP was used to prepare PES substrates via a phase inversion process. Briefly, the polymer solution was cast on a clean flat glass plate by using a casting knife with 100 µm in height, followed by immersing the glass plate immediately in a DI water coagulation bath at room temperature. A PES substrate consisting of a dense layer and a porous support was formed instantly and peeled off the glass plate in a couple of minutes. The PES substrate was then transferred to another water bath for complete solvent exchange and stored in fresh DI water for further experiments [26].

A nascent FO membrane was synthesized via the interfacial polymerization between MPD and TMC occurred on the dense surface of a PES substrate according to an established approach. First, the PES substrate was immersed in a 2.0 wt% MPD solution for 2 min, then taken out from the solution and cleaned with a filter paper. The MPD-saturated PES substrate was subsequently soaked in n-hexane containing 0.15 wt% TMC for 1 min. The PA layer was synthesized via an amidation reaction between MPD and TMC on the PES substrate. The resultant nascent FO membrane was left for further modification or stored in DI water for subsequent characterization and FO experiments [26].

### 2.3. Preparation of the Arg-PES FO Membrane

The novel Arg-PES FO membrane was developed by grafting Arg onto the PA layer of a nascent FO membrane. This is accomplished via an amidation reaction between the amine groups within arginine and acyl chloride groups in the PA layer. Specifically, to determine the optimal reaction conditions, the nascent FO membrane was immersed in Arg solution with concentrations from 0.5, 1.0, 2.0, 3.0 wt%. Varying reaction time intervals of 5, 10, 20, and 30 min were conducted to explore the optimal reaction conditions. Subsequently, excess arginine solutions were then removed and washed with DI water. The resulting Arg-PES FO membranes were stored in water for further experiments.

### 2.4. Characterization of FO Membranes

Physicochemical and Morphological Properties. The physicochemical and morphological properties of FO membranes, including the surface functional groups, hydrophilicity, zeta potential, morphology, and topology, were characterized systematically with membrane samples dried by a freeze dryer (ModulyoD, Thermo Electron Cor., Waltham, MA, USA) for overnight at −50 °C. The functional groups on membrane surfaces were analyzed by attenuated total reflection Fourier transform infrared spectroscopy (ATR-FTIR, Prestige-21, Shimadzu, Kyoto, Japan) in the range between 400 cm^−1^ and 1800 cm^−1^. The surface hydrophilicity was evaluated by dynamic water contact angle measurements conducted on a goniometer (Precise Test, Dongguan, China) at room temperature. A 2 μL DI water droplet was dropped onto the membrane surface and kept for 5 s followed by image capture and contact angle calculation by CAM2008 software version 1.0. The zeta potential of membrane surfaces was determined by a surface zeta potential electrode assembly (SZP, Brookhaven Instruments, NanoBrook Omni, Holtsville, NY, USA). The samples were immersed in a 1.0 mM KCl solution with pH = 7.0 at 25 °C in the course of experiments. Membrane morphology and topology were analyzed separately by a field emission scanning electron microscope (FESEM, Nova NanoSEM 230, FEI, Long Island, NY, USA) and an atomic force microscope (AFM, Agilent, Santa Clara, CA, USA) from Digital Instrument.

Structural Properties. Membrane structural properties, including MWCO, pore size, and pore size distribution, were characterized by the solute separation approaches. Membrane rejections to a range of neutral solutes with different molecular weights at 200 ppm solutions were investigated through a bench-scale dead-end filtration system. The salt rejection ratio *R* (%) was then obtained from Equation (1):

(1) $$R=(1-\frac{C_{p}}{C_{f}})\times 100\%$$

where *C_p_* (mol·L^−1^) and *C_f_* (mol·L^−1^) are the concentrations of permeation and feed solutions, respectively, which were measured by a total organic carbon analyzer (TOC-L CSH ASI, Kyoto, Japan).

The structural properties of PES substrates were determined based on the rejections to PEG with MW = 12,000 and 35,000 g·mol^−1^, and PEO with MW = 100,000 and 200,000 g·mol^−1^. While those of the FO membranes were evaluated after knowing the rejections of ethylene glycol, diethylene glycol, triethylene glycol, and glucose solutions at 200 ppm according to the literature method [27,28].

Mass Transfer Properties. Factors indicating membrane mass transfer properties, such as the water permeability coefficient (*A*, LMH·bar^−1^), salt permeability coefficient (*B*, LMH), and salt rejection (*R*, %), were evaluated by testing the membrane in an RO process. The experiments were carried out under a 2.0-bar pressure at room temperature against DI water with an effective membrane area of 3.14 cm^2^. The membrane water permeability coefficient can then be determined using Equation (2):

(2) $$A=\frac{V}{A_{m}\cdot ∆t\cdot ∆P}$$

where *V* (L) is the water volume on the permeate side collected over time of Δ*t* (h), *A_m_* (m^2^) is the effective membrane area, and Δ*P* (bar) is the applied pressure.

Membrane salt rejection was evaluated by the rejection of NaCl at 500 ppm. The conductivities of the feed and permeate were measured by a calibrated conductivity meter (REX, DDSJ-308F). The salt concentrations on the feed (*C_f_*, mol·L^−1^) and permeate sides (*C_p_*, mol·L^−1^) were then obtained, respectively, from the standard curve developed from the relationship between the solution conductivity and known concentration. The NaCl rejection can, thus, be calculated by Equation (1).

Salt permeability coefficient (*B*, LMH) was obtained based on the relationship with the water permeability coefficient and rejection as described in Equation (3), according to the solution-diffusion theory:

(3) $$B=\frac{1-R}{R}A(∆P-∆\pi )$$

where Δ*π* (bar) is the osmotic pressure differential across the FO membrane.

### 2.5. FO Process

The impacts of Arg as a membrane modifier on performance were systematically evaluated in FO separation processes. FO experiments were operated through a membrane module with a membrane area of 4.5 cm^2^ and the solutions on both sides were flowing counter-currently at 1.3 cm·s^−1^ at 25 ± 0.5 °C. Performance under the pressure retarded the osmosis (PRO) (feed solution against the support layer) mode and FO (feed solution against the selective layer) mode, which were both measured in FO experiments [27,28].

The FO water recovery efficiency was evaluated by water flux, *J_w_* (LMH), which was determined by the weight increase of the draw solution per unit area and time, as described in Equation (4):

(4) $$J_{w}=\frac{∆m}{A_{m}\cdot ∆t\cdot \rho }$$

where Δ*m* (kg) is the weight change of the draw solution during an interval of Δ*t* (h), and *ρ* (kg·L^−1^) is the DI water density.

The reverse solute flux of draw solution, *J_s_* (gMH), was calculated from Equation (5) based on the concentration changes of feed solution before and after experiments [8]:

(5) $$J_{s}=\frac{\left(C_{t}V_{t})-(C_{0}V_{0}\right)}{A_{m}\cdot ∆t}$$

where *C*_0_ (g/L) and *V*_0_ (L) are the respective initial concentration and feed volume; *C_t_* (g/L) and *V_t_* (L) are the respective final concentration and volume of the feed solution.

### 2.6. Antifouling Performance

A lab-scale FO system was employed to investigate membrane performance with respect to dewatering oil/water emulsion solutions (surfactant/petroleum ratio of 1/9 (wt%)). The short-term membrane fouling tests were performed for water reclamation 0–3000 ppm oil-in-water emulsions stabilized by Tween 80, SDS, and CPC, respectively, for 10 min. Long-term tests for water reclamation 1000 ppm oil/water emulsions stabilized with Tween 80, SDS, and CPC were investigated over a 12 h period. Membrane flux recovery was evaluated in long-term experiments, where the membrane was cleaned with DI water every 12 h. All experiments were conducted with a 2.0 M NaCl draw solution at neutral and ambient conditions, if not otherwise specified.

### 2.7. Antibacterial Property Evaluation

The antibacterial properties of the modified and unmodified membranes were evaluated by the *E. coli* colony counting method (CC). Simply cut the membranes into small pieces, approximately 1 cm × 3 cm in size, immersed them in deionized water, and soak them for a while. Following drying, they are transferred to a sterile operating table and sterilized under a UV lamp for 15 min. An appropriate dilution of 0.1 mL bacterial solution (about 10^6^ cfu/mL) was prepared and coated onto a plate; and then the modified membrane and the PA membrane were placed on an appropriate solid LB agar medium in order, with the active surface layer in contact with the medium. The plate was inverted and placed in a constant temperature incubator at 37 °C for 12 h, and the growth of bacteria was observed. The number of *E. coli* colonies grown on solid media with or without modified membrane was recorded as *C_a_*. Additionally, the bacterial solution medium (without membrane) was used as a blank sample, and the number of *E. coli* colonies was recorded as *C_b_*. The antibacterial efficiency of membrane was calculated according to the (*E_b_*) Equation (6) [29,30]:

(6) $$E_{b}=(1-\frac{C_{a}}{C_{b}})\cdot 100\%$$

## 3. Results and Discussion

Membrane fouling is the main obstacle in membrane-based water treatment, caused by the deposition of macromolecular pollutants or microorganisms on the membrane surface, resulting in reversible or irreversible contamination of the membrane. To alleviate this phenomenon, a modifier is used to improve the hydrophilicity or antimicrobial properties of the membrane. In this study, we grafted a zwitterionic Arg onto the membrane surface through a rapid and efficient amidation reaction. The desired Arg-PES membrane was formed on the PA-PES membrane, giving it high hydrophilicity and antibacterial properties, while maintaining excellent fouling resistance and ease of cleaning for sustained and efficient water-recovery efficiency. This makes it an ideal material for antifouling and antibacterial purposes.

### 3.1. Preparation and Characterizations of FO Membranes

**Figure 2:**
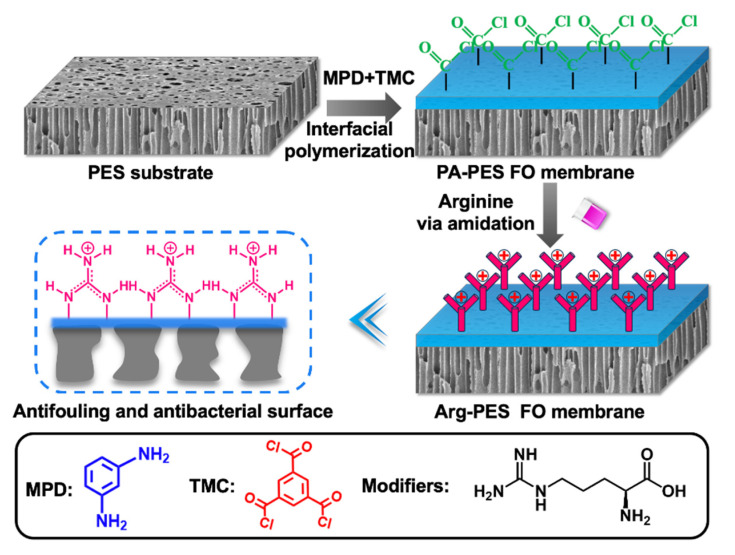
The process of membrane preparation.

Prior to synthesizing the modified membrane, as illustrated in Figure 2, a nascent FO membrane composed of a PES substrate and a PA selective layer was prepared. The porous PES substrate was prepared by the phase inversion method. TMC and MPD undergo an amidation reaction on the PES substrate to form a dense PA layer and create the nascent FO membrane. There are numerous unreacted TMC acid chloride groups [31]. As reaction sites, they serve for rapid and mild amidation reactions with a primary amine on the arginine modifier, ultimately grafting onto the membrane. Once grafted onto the membrane, the unique nature of the guanidine group on the arginine facilitates the formation of a unique rigid structure on the membrane surface.

**Figure 3:**
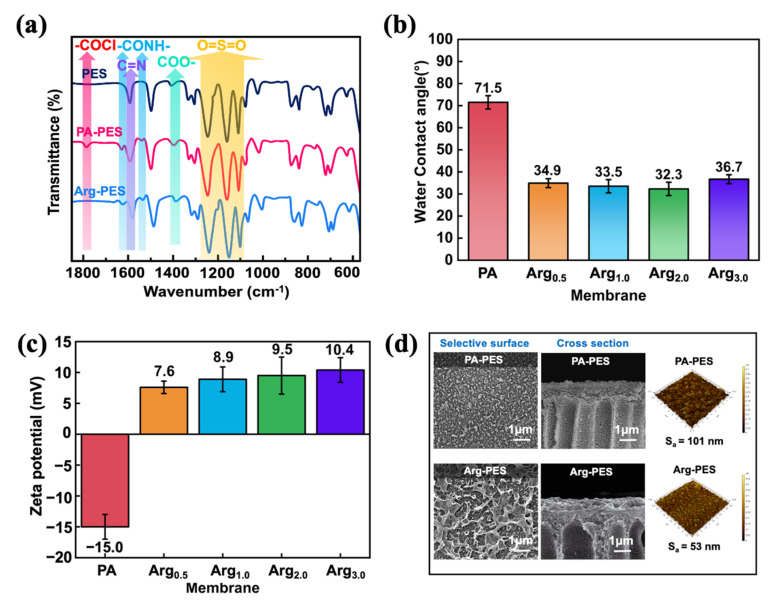
(**a**) FTIR spectra of PA-PES and modified PA-PES membrane; (**b**) water contact angles of PA-PES, modified PA-PES FO membranes; (**c**) zeta potential of PA-PES and modified PA-PES membrane; (**d**) morphological and topological properties of the membrane: SEM images and AFM images.

As shown in Figure 3a, FTIR spectra are commonly used to characterize the chemical properties of the membrane surface. O=S=O corresponding to PES appears at 1242, 1106 cm^−1^. Compared to the PES support layer, the PA-PES composite FO membrane shows new absorption peaks at 1786 (COCl), 1632, and 1531 cm^−1^ (CONH), which are the characteristic peaks of the PA layer. This indicates that the PA layer was successfully deposited onto the PES support layer [31]. After modification with the zwitterionic Arg, COCl disappeared and characteristic peaks of carboxy COO (1390 cm^−1^) and guanidinium C=N (1596 cm^−1^) appeared [25]. From the above results, it can be known that the zwitterionic Arg was successfully grafted on the membrane.

It can be seen from the water contact angle test in Figure 3b that the zwitterionic Arg modifier can effectively reduce the water contact angle on the membrane surface after modification, indicating increased hydrophilicity. The presence of carboxyl and guanidine groups on the modified membrane significantly enhances its hydrophilicity, as compared to unmodified membranes [32,33]. These are not available in the PA-PES membrane. It should note that the hydrophilicity of the membrane surface correlates directly with water permeability. Thus, increasing hydrophilicity typically leads to higher water permeability. This observation aligns well with experimental results obtained from water permeability characterization.

To ascertain the effect of the modifiers on the membrane surface charge, we systematically tested the zeta potential of both unmodified and modified membranes. As depicted in Figure 3c, the surface of the PA-PES FO membrane carries a negatively charged due to the hydrolysis of the acid chloride group into carboxyl group. After grafting the modifier, amine, and guanidine groups from the Arg modifier, they covalently bind with the acidic chloride on the membrane surface. This leads to a reduction in carboxylic acid dissociation and results in a positive membrane potential. Additionally, numerous guanidine groups present on the modifier contribute to generating special stability as they are strong electron donors with effects similar to benzene rings. Ultimately, dominant positively charged guanidine groups impart a strong positive charge to the membrane surface. These changes in surface charge indicate successful grafting of modifiers onto the membrane surface.

### 3.2. The Morphological and Structural Properties of FO Membranes

Morphological observations were conducted using SEM. Figure 3d illustrates the surface and cross-sectional morphology of unmodified and modified membranes. On the surface of the dense PA membrane, COCl in TMC reacts with NH_2_ in the modifiers to form a loose modified layer. It is worth noting that the Arg-PES membrane has a more critical repellent layer with a larger specific surface area. This modified layer can reduce the surface energy of the membrane, thereby weakening the adhesion of contaminants on the membrane surface [34]. It also aids in the removal of the contaminants deposited on the membrane surface by the water shear force. Overall, this modified layer effectively prevents the adhesion of contaminants on the membrane surface.

To analyze the surface morphology and roughness in depth, an AFM was utilized. It is evident that the roughness of the modified membrane has decreased in comparison to the PA-PES membrane. This phenomenon could be attributed to the improved maneuverability of the modified substance. As a result of their heightened reactivity and fluidity when reacting with acid chloride groups, the Arg-PES membrane possesses denser and smoother surfaces, with correspondingly lower S values. In summary, smoother modified membranes are prepared, which is beneficial to reduce the adsorption of pollutants on the membrane surface.

**Figure 4:**
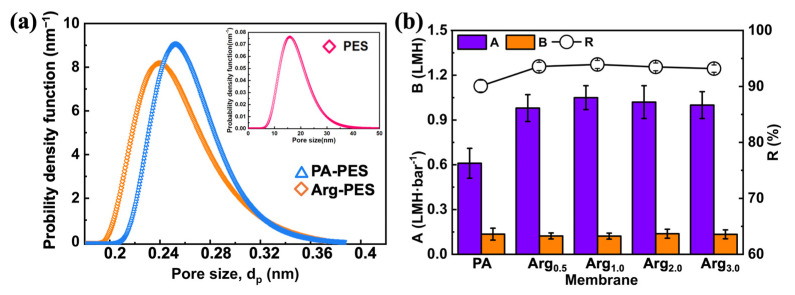
Structural and water transfer properties: (**a**) pore size and pore size distribution; (**b**) values of A, B, S, R.

To demonstrate the variations in the membrane structure and mass transfer characteristics, resulting from modification, we systematically investigated the pore structure, as well as the mass transfer coefficients for water flux and reverse salt flux in both the modified and the unmodified membranes. The PA-PES membrane is prepared by coating the PA layer onto a PES support structure, which reduces pore size to the FO level. Similar to the interfacial polymerization process, chemical modification can also alter pore size distribution. Figure 4 illustrates the pore size distributions, structure, and mass transfer characteristics of both the PA-PES membrane and its modified counterparts. It indicates that the Arg-PES membrane has smaller pore sizes than the unmodified membrane. The surface density was further enhanced, while the average pore diameter and relative cut-off molecular weight decreased from 0.26 nm to 0.24 nm. This suggests that the guanidine modifiers did not compromise the structure of the membrane and, at the same time, provided an additional layer to form a denser surface.

The mass transfer parameters of membranes are also shown in Figure 4. Compared with the PA-PES FO membrane, the modified membrane possesses a denser surface and unique hydrophilic functional groups, resulting in higher salt rejection. While the PA-PES FO membrane had a NaCl rejection rate of 90.07%, the Arg-PES membrane exhibited rejections of 93.89%, respectively. The improvement in rejection is beneficial in reducing the reverse salt flux and maintaining effective osmotic pressure on both sides of the membrane. Additionally, it is noteworthy that the water permeability coefficient of the modified membrane has increased, while the density of membrane has improved. This improvement in water permeability is attributed to the incorporation of hydrophilic functional groups, such as guanidine groups and carboxylic acid groups etc., which enhance surface hydrophilicity and significantly improve the water permeability efficiency.

### 3.3. Separation Performance of FO Membranes

**Figure 5:**
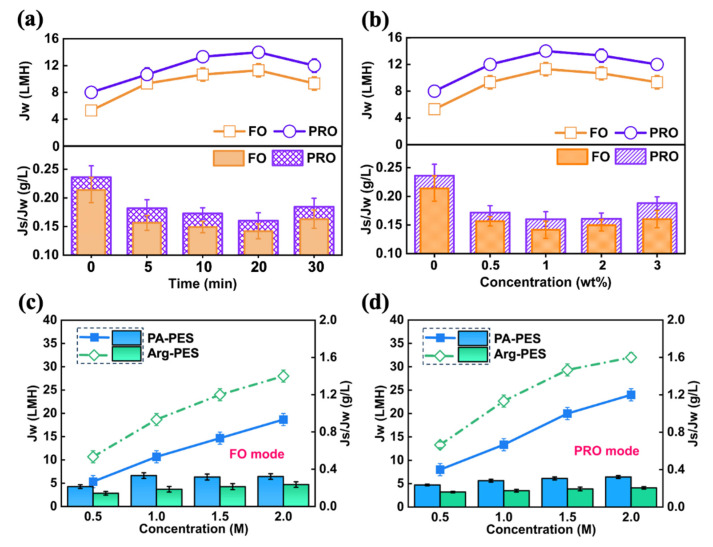
Modified Arg-PES membrane with (**a**) optimal modification time and (**b**) concentration conditions; and exploration of different concentration of NaCl draw solution (**c**) FO mode, (**d**) PRO mode.

The experimental conditions utilized for membrane modification play a significant role in determining its performance. We systematically investigated the synthesis conditions of the modified membrane, varying the reaction time and modifier concentration to obtain optimal separation performance. The experimental results show that variations in both the reaction time and concentration of the modifier lead to similar trends in water flux trends. The trade-off between membrane hydrophilicity and mass transfer resistance results in a tendency for water flux, as illustrated in Figure 5a,b. The trend of water flux over time corresponds to the concentration of the modifier used. Specifically, within a given timeframe, increasing the modifier concentration initially leads to a boost in water flux and then gradually diminishes. As additional modifiers are attached to the membrane surface, its hydrophilicity augments, resulting in better water permeability. However, concurrently, increasing modifier concentration also elevated the thickness of the membrane, thereby amplifying the resistance to water permeability, and subsequently diminishing water permeability.

The optimum modification conditions depend on the hydrophilic nature of the modifier etc. The optimum modification conditions for Arg-PES membranes were 20 min at 1.0 wt%. Under these conditions, Arg-PES membranes exhibited a 75.0% higher water flux in PRO mode and 113.2% higher in FO mode than PA-PES membranes, while also significantly lowering reverse salt flux [35].

A comprehensive evaluation of the modified membrane was conducted, as shown in Figure 5c,d, by altering the membrane orientation and the draw solution concentration. Baseline studies were also carried out on the nascent PA membrane. Regardless of membrane orientation, the water flux increases linearly with the draw solution concentration. Higher solution concentration led to an increase in osmotic pressure difference, driving force across the membrane, and water permeability. However, due to the detrimental effects of internal concentration polarization (ICP), PRO mode consistently outperformed FO mode for all membranes [36]. Grafting of the modifier proved to be beneficial to the separation process, resulting in higher water flux and less solute loss than the unmodified membrane.

When 0.5–2.0 M NaCl was used as the draw solution, the water flux of Arg-PES membranes increased by 33.3–66.3% (PRO mode) and 50.9–100.1% (FO mode), compared to PA-PES membrane. The possible reason for this improvement is the large number of guanidine groups on the membrane surface, which form special structures. This increases the specific surface area of the membrane and provides continuous hydration for the transfer of water molecules, thereby enhancing the water diffusion rate through the membrane.

In general, the Arg-PES membrane exhibited superior performance in this study. It is worth noting that the performance of this type of membrane is also comparable or better than other recently reported FO membranes. Table 1 offers a comparison of FO performance, such as 2-[(2-aminoethyl) amino]-ethane sulfonic acid monosodium salt (SEA) modified membrane, 1,4-bis(3-aminopropyl) piperazine propane sulfonate (P-2SO_3_-2NH_2_) modified membrane, n-aminoethyl piperazine propane sulfonate (AEPPS) modified membrane, (1-(3-aminopropyl)-imidazole) propane-sulfonate (APIS) modified membrane, and quaternization 2,6-diaminopyridine (QDAP) modified membrane [17,26,37,38,39].

We posit that a combination of factors contributes to the remarkable water permeability, minimal salt diffusion, and overall performance of the Arg-PES membrane. This is a synergistic relationship. By virtue of a straightforward, mild, and efficient amidation reaction, a significant quantity of arginine is successfully grafted onto the membrane surface. This imparts high hydrophilicity to the membrane surface while maintaining a stable hydration layer. It reduces reverse salt flux while maintaining water flux. Notably, the potent positive charge, coupled with its smooth surface characteristics, further amplifies the reduction in salt flux, thereby underpinning the overall performance of the Arg-PES membrane. These properties make it a promising candidate for various water treatment applications.

**Table 1:** FO performance comparisons between the Arg-PES membrane and other recently-developed membranes.

FO Membrane	Water Flux (LMH)		Reverse Salt Flux (gMH)		*J_s_*/*J_w_* (g/L)		Draw Solution (M)	Refs.
	FO	PRO	FO	PRO	FO	PRO		
Arg-PES	22.7	26.7	3.1	3.5	0.14	0.13	1.0	This work
SEA-PES	13.5	-	8.8	-	0.65	-	1.0	[37]
P-2SO_3_-2NH_2_-PES	14.7	20.0	4.4	5.0	0.3	0.25	1.0	[17]
AEPPS-PES	15.0	27.0	5.7	11.3	0.38	0.42	1.0	[38]
APIS-sPES	22.7	26.0	3.4	3.6	0.15	0.14	1.0	[26]
QDAP-PES	25.1	-	4.0	-	0.16	-	1.0	[39]

Note: DI water and NaCl are the respective feed and draw solutions. SEA: 2-[(2-aminoethyl) amino]-ethane sulfonic acid monosodium salt, P-2SO_3_-2NH_2_, AEPPS: n-aminoethyl piperazine propane sulfonate, APIS: (1-(3-aminopropyl)-imidazole) propane-sulfonate, QDAP: quaternization 2,6-diaminopyridine, PES: polyethersulfone.

### 3.4. Water Reclamation from Oil/Water Emulsions via FO Membranes

**Figure 6:**
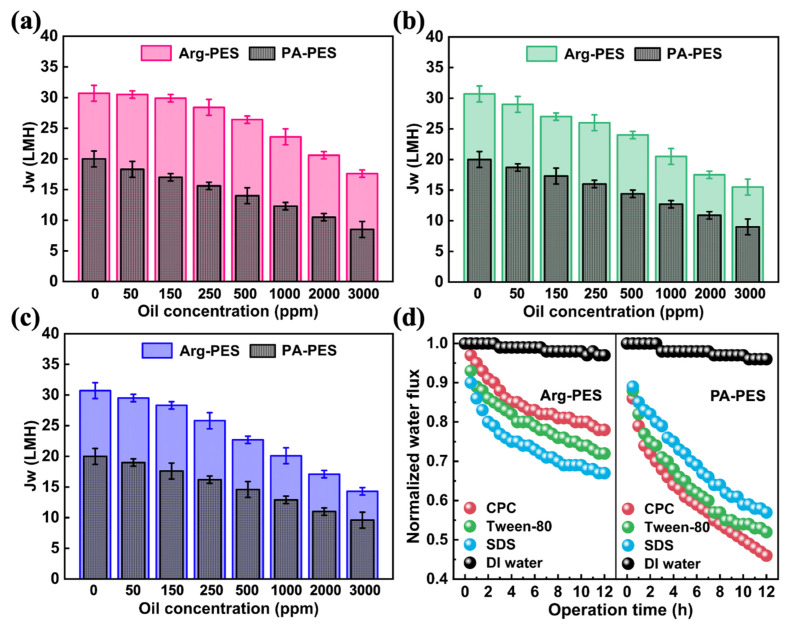
Comparison of short-term performance between the nascent membrane and modified membrane in oil–water separation with (**a**) CPC (short-term), (**b**) Tween-80 (short-term), (**c**) SDS (short-term), and (**d**) long-term.

We evaluated the antifouling performance of the optimal-modified Arg-PES and PA-PES membranes for oily wastewater treatment. In FO mode, water was recovered from the emulsified oil with concentrations ranging from 0–3000 ppm, the oil droplets sized between 200–1000 nm, and operating times ranging from 10–720 min. As shown in Figure 6, an increase in feed concentration led to higher osmotic pressure on this side, and a decrease in net osmotic pressure on both sides, causing a reduction in water permeability. Additionally, an increase in feed concentration caused the solution to become more viscous, leading to membrane fouling and decreased water flux.

In short-term experiments, the Arg-PES membranes outperformed the PA-PES membranes in terms of water flux when processing positively charged emulsified oils (CPC) by 53.5–96.5%, as depicted in Figure 6a; neutral emulsified oils (Tween-80) by 53.5–66.7%, as illustrated in Figure 6b; and negatively charged emulsified oils (SDS) by 53.5–60.8%, as shown in Figure 6c. As the pores of the FO membrane are around 0.1 nm, which is much smaller than oil droplet size, their rejection rates were all above 99.9%.

From long-term experiments shown in Figure 6d, it demonstrates that the Arg-PES membrane had better FO performance than the PA-PES membrane when processing three different types of emulsified oil. The specific values are 30% (CPC), 19% (Tween-80), and 9% (SDS). This is because Arg-PES membranes can form hydration layers that greatly enhance water permeability. Specifically, the “structure-breaking” effect of the guanidine group splits the large water molecule clusters around the guanidine group into smaller clusters, allowing water molecules to permeate through the membrane channels [33]. Furthermore, the high hydrophilic surface energy maximizes surface hydration, which effectively weakens interactions with oil contaminants, reducing deposition and adsorption, while improving both water permeability efficiency and antifouling capability. Additionally, the larger specific surface area provided by modified surfaces offers continuously hydrated pathways for transferring water molecules.

Meanwhile, electrostatic forces at work affect separation performance for emulsifiers. Under CPC-emulsified oil conditions, Arg-PES membranes have repulsive effects on oil contaminants, whereas PA-PES membranes exhibit adsorptive effects that weaken FO performance; under SDS-emulsified oil conditions, Arg-PES membranes attract contaminants, leading to reduced water flux; and under Tween-80-emulsified oil conditions, the most severe drop in water flux occurs due to its smaller particle size, causing hydrogen bonding interactions with the membrane surface, affecting its antifouling properties [40]. It is worth noting that surfactant–membrane interactions, in addition to the type of surfactant used, also influence separation performance in the FO process.

**Figure 7:**
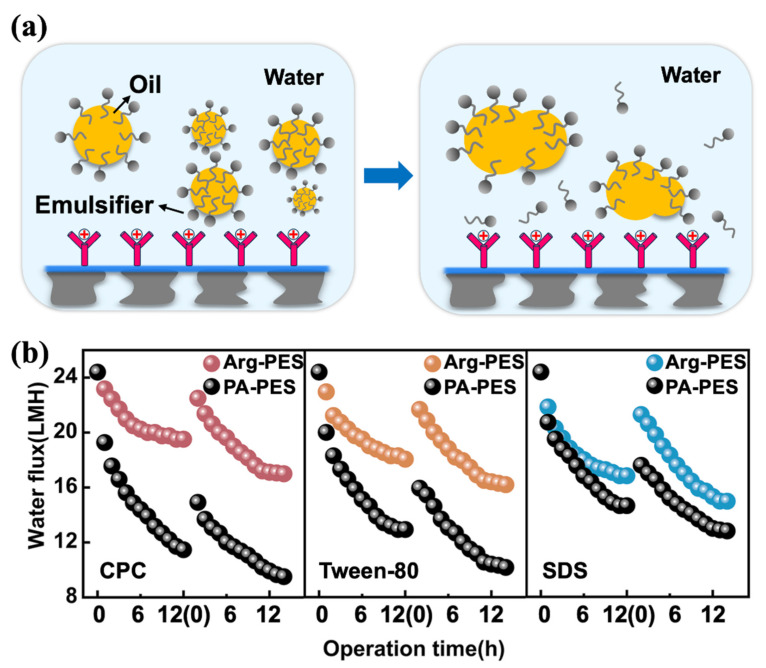
(**a**) The demulsification mechanism, (**b**) comparison of the nascent membrane and modified membrane regeneration properties.

To clarify the interaction between the modified membrane surface and various oil droplets, we present a detailed explanation in Figure 7a. The emulsification effect is deemed to be promoted by the strong electrostatic interaction between the Arg-PES membrane and oil droplets. This demulsifying effect occurs by either a bridging or charge replenishment mechanism [40]. As charged oil droplets approach the membrane, electrostatic attraction or repulsion causes the surfactants to migrate on the surface of oil droplets [41]. In the case of positively charged Arg-PES membranes, electrostatic attraction causes the migration of anionic SDS surfactants towards the membrane side, increasing the surface tension at the top of oil droplets and promoting aggregation into larger sizes. Conversely, cationic CPC surfactants in emulsified oils are repelled by positive membranes and unevenly distributed, leading to further aggregation of oil droplets. Therefore, we reckon that the mechanism of emulsion oil separation is the uneven distribution of surfactants on the surface of oil droplets and their agglomeration effect, which promotes the demulsification process [42,43]. Based on this mechanism, strongly positive surfaces are conducive to efficient demulsification and, thus, beneficial for emulsion separation. This suggests that there are two competing factors affecting emulsion oil separation performance: the charge interaction and the emulsion oil droplet size. When the charge interactions are not significant, the particle size of the emulsion oil droplets plays a critical role in the formation of oil contamination.

We further investigated the reversibility of membrane fouling in FO mode. Following every 12 h emulsion oil separation, a 30 min membrane cleaning cycle was conducted. It can be seen from Figure 7b that both PA-PES and Arg-PES membranes were able to effectively recover some water flux after cleaning, indicating that oil fouling is reversible in FO mode. In the first cycle, for Arg-PES membranes contaminated with CPC emulsified oil, Tween-80 emulsified oil, and SDS emulsified oil, the water flux was restored to 92%, 87%, and 86% of its initial value, respectively, while for PA-PES membranes it was only restored to 61%, 65%, and 72%. It is evident that the modified membrane has potent renewability against all types of emulsified oil.

It is noteworthy that in the case of SDS emulsified oil contamination, the water flux recovery capacity of both types of membranes increased, which is associated with its hydrophilic–lipophilic balance (HLB). Despite negatively charged SDS emulsified oil being attracted to the modified membrane surface, differences in the HLB values of the surfactants (SDS = 40, CPC = 12, Tween = 10) can also affect separation performance. The higher the HLB value, the better the hydrophilicity it possesses. Hence, even if SDS molecules aggregate on the Arg-PES membrane surface during the separation process, they can be easily washed away by water. A similar phenomenon was observed in the PA-PES membrane. The high reversibility of the Arg-PES membrane to CPC emulsified oil, Tween-80 emulsified oil, and SDS emulsified oil is mainly attributed to (1) a strongly positively charged membrane surface, (2) a relatively large specific surface area, and (3) the high hydrophilicity of guanidine groups.

### 3.5. Antibacterial Property Evaluation of FO Membranes

**Figure 8:**
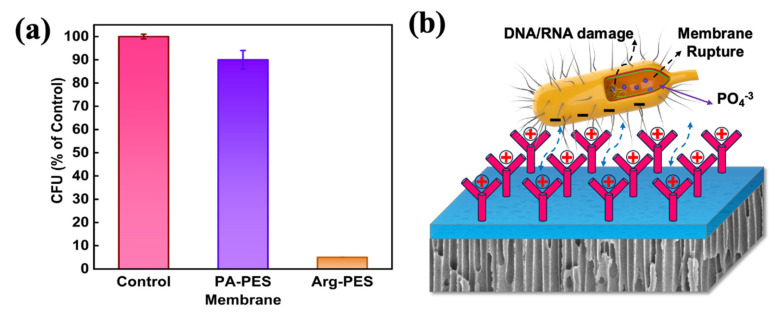
(**a**) Total number of bacterial colonies after treatment with control group, PA-PES, and Arg-PES membrane; (**b**) schematic of the possible interaction between the *E. coli* and Arg-PES membrane.

The antibacterial properties of PA-PES and Arg-PES membranes were evaluated using the traditional *E. coli* colony counting method [44]. The diluted bacterial solution was inoculated on an agar plate and contacted with the active layer side of the membrane for 12 h, after which viable cells count was performed to evaluate the antibacterial properties of the membrane. Figure 8a demonstrates that the culture medium for the nascent membrane exhibited a substantial number of *E. coli* colonies, whereas the culture medium for the modified membrane had only a few colonies. Generally, a live *E. coli* cell is capable of forming colonies when cultured on a solid medium. Thus, based on the number of *E. coli* colonies present on the culture medium, the antibacterial rate of the nascent membrane was calculated to be approximately 10%, whereas that of the modified membrane was approximately 96%.

Figure 8b delineates the antibacterial mechanism of the Arg-PES membrane. The positively charged guanidine groups establish robust hydrogen bonds with the negatively charged phosphates present on the bacterial cell membrane [33,45]. This interaction induces perturbation in the phospholipid bilayer, ultimately leading to membrane rupture. Furthermore, the guanidine cations effectively target the pores within the bacterial outer membrane, precipitating the loss of essential nutrients (DNA/RNA) and water from the bacterial structure. Consequently, this process results in bacterial contraction and eventual demise.

## 4. Conclusions

In response to the existing research gaps in targeted oil–water separation, we addressed the concerns of elevated pollution and biological contamination within the FO membrane. This work aimed to improve the antifouling performance and antibacterial properties of PA-PES FO membranes by grafting highly hydrophilic zwitterionic Arg via an amidation reaction, resulting in the creation of Arg-PES membrane. The modified Arg-PES membrane distinctly exhibits an augmented hydrophilic surface, coupled with lower roughness and a positive charge, compared to the PA-PES membrane. This modification led to superior antifouling capacity against emulsified oil. This manifests a lower flux decline rate, higher flux recovery rate, and enhanced membrane renewability, especially when treating CPC emulsified oil during oily wastewater treatment. Furthermore, the Arg-PES membrane demonstrated robust antibacterial properties against *E. coli*.

Overall, this study provides an effective strategy for designing FO membranes, harnessing the potential of arginine, for the creation of formidable antifouling and antibacterial attributes. This strategic approach offers valuable references and insights that hold the potential to inform the scalability and real-world applicability of managing oily wastewater in practical scenarios.

## Data Availability

Not applicable.
